# Seafood consumption and umbilical cord blood mercury concentrations in a multiethnic maternal and child health cohort

**DOI:** 10.1186/1471-2393-14-209

**Published:** 2014-06-18

**Authors:** Reni Soon, Timothy D Dye, Nicholas V Ralston, Marla J Berry, Lynnae M Sauvage

**Affiliations:** 1 Department of Obstetrics, Gynecology, and Women’s Health, University of Hawaii, 1319 Punahou Street, Suite 824, Honolulu 96826, Hawaii; 2 Department of Obstetrics and Gynecology, University of Rochester, 265 Crittenden Boulevard, CU 420708, Rochester 14642-0708, New York; 3 Energy & Environmental Research Center (EERC), University of North Dakota, 15 North 23rd Street, Stop 9018, Grand Forks, North Dakota 58202-9018; 4 Department of Cell and Molecular Biology, University of Hawaii, 651 Ilalo St, Room 222G, Honolulu 96813, Hawaii

**Keywords:** Mercury, Fish, Seafood, Pregnancy, Hawaii, Nutrition, Environment

## Abstract

**Background:**

Fish consumption is common among the cultures of Hawaii, and given public health attention to mercury exposure in pregnancy, it is important to better understand patterns of fish consumption and mercury in pregnancy. This study examined the influence of maternal fish consumption during pregnancy on umbilical cord mercury (Hg) concentrations in a multiethnic cohort of women in Hawaii.

**Methods:**

This secondary analysis of a prospective cohort pilot study examined antenatal seafood consumption and neonatal outcomes in Hawaii. The first 100 eligible women who consented were enrolled. After delivery, umbilical cord blood and a dietary survey were obtained.

**Results:**

Most women (86%) consumed seafood during the month prior to delivery. Overall, 9% of women consumed more than the recommended limit of 12 ounces/week. Seafood consumption varied significantly by ethnicity and income, with 30% of poor women consuming more than the recommended limit. Seafood consumption did not vary by age or education.

Umbilical cord blood Hg levels were 5 μg/L or more in 44% of women. Filipina were significantly less likely to have elevated Hg levels compared with non- Filipina (p < .05). Mercury levels did not vary by other demographic characteristics.

Women reporting consumption exceeding 12 ounces fish per week were significantly more likely to have cord blood Hg levels of 5 μg/L or more, but mean Hg concentrations were not significantly higher (6.1 ± 3.3 v 5.0 ± 3.7). The odds ratio for elevated Hg, however, was significant among seafood-consumers compared with non-consumers (5.7; 95% confidence interval: 1.2, 27.1).

**Conclusions:**

Despite Environmental Protection Agency (EPA) guidelines, a significant portion of pregnant women consumed more than the recommended amount of seafood, which was associated with race and income. Further, almost half of study participants had cord blood Hg concentrations at or exceeding 5 μg/L.

## Background

Methylmercury (MeHg) toxicity became known after incidents in Japan and Iraq involving exceptionally high-level human exposure resulted in severe neurological deficits in fetuses born to mothers who were among the exposed [1,2]. Consumption of fish and other seafoods are the dominant sources of MeHg exposure for most people [3]. While high MeHg exposures have been shown to cause significant neurological deficits, whether or not the lower MeHg exposures, typically experienced by most people, result in any adverse effects remains unclear. Prenatal and postnatal exposures have been associated with adverse neurodevelopmental effects, however the results are conflicting. In the Faroe Islands cohort, children with a mean cord blood Hg of 22.9 μg/L were found to have Hg-associated deficits in the domains of language, memory, and attention at 7 years of age [4] that were persistent at 14 years of age [5]. Additionally, a study in New Zealand found decreased performance on scholastic and psychological tests associated with high prenatal MeHg exposure [6]. In contrast, in the Seychelles study cohort, higher prenatal and postnatal MeHg exposures were not associated with adverse effects in children followed out to 17 years, with beneficial associations being repeatedly noted instead [7]. Additionally, mercury exposure levels below those originally identified as potentially harmful to child and infant health have also shown negative health impacts [8]. The potential negative effects of mercury exposure and the protective effect of fish consumption confound understanding research in this area [9].

Based primarily on cord blood levels found in the Faroe Islands study [10], the Environmental Protection Agency (EPA) and the National Research Council (NRC) determined a benchmark dose lower limit (BMDL), or the lowest dose expected to be associated with a small increase in the incidence of adverse outcome, of 58 μg/L (ppb) MeHg in cord blood [11]. As a base to calculate a reference dose, or maximum acceptable dose of a toxic substance, the NRC recommended adding an uncertainty factor of 10 to this estimate, because of biological variability and inadequate data [11]. The EPA agreed and established a reference dose of 5.8 μg/L MeHg in blood in their recommendations to limit exposures of pregnant and nursing mothers and young children [12]. The 2004 EPA and Food and Drug Administration (FDA) advice for these vulnerable populations are to avoid shark, swordfish, king mackerel, and tilefish, but to eat up to 12 ounces a week of fish with low mercury contents [13].

Complicating this policy recommendation, however, is the well documented evidence that seafood that is high in omega-3 fatty acids is associated with enhanced infant and child neurodevelopment [14-17] and that MeHg developmental neurotoxicity may be confounded by its physiologic interrelationships with the trace element selenium [18,19]. Children whose mothers consume less seafood during their pregnancy may in fact be at higher risk for abnormal neurodevelopment [17].

Research looking into the interactive roles of mercury, selenium and omega three fatty acids on fetal and infant neurodevelopment is scarce [20]. Hawaii is home to a multiethnic population and cultures for which seafood consumption is an essential part of historical and present-day life. A previous study done in Hawaii found 17.6% of participants consumed more than the recommended amount of seafood and significantly more of these women had elevated cord blood mercury concentrations when compared with women who stayed within the guidelines [21]. Mean MeHg cord blood concentrations were 4.82 μg/L, with a high of 20 μg/L. In their report to the NRC, the Committee on the Toxicological Effects of Methylmercury issued a public health recommendation to study fish consumption in various subpopulations to identify those at higher risk for adverse mercury related effects [11].

A pilot study was conducted to evaluate umbilical cord blood concentrations of mercury, selenium and omega three fatty acids and various neonatal outcome measures. This present report is a secondary analysis, with the primary hypothesis of interest being that there is a positive correlation between dietary recall of seafood consumption and umbilical cord blood concentrations of mercury. The secondary hypothesis is that dietary intake of fish during pregnancy varies by ethnic and demographic subpopulations.

## Methods

Participants were enrolled into the primary study when they presented for admission to the Labor and Delivery Suite at Kapiolani Medical Center for Women and Children in Honolulu, Hawaii between June 2010 and March 2011. Kapiolani Medical Center is the largest birthing hospital in Hawaii, accounting for a large proportion of the state’s births, particularly those within Oahu. Women were considered eligible if they were 18 to 45 years old and pregnant with a term singleton fetus. Women with chronic medical conditions or who reported tobacco, alcohol, or illicit drug use were excluded. Patients who met eligibility criteria were approached about participating in the study. Because specimens had to be processed within two hours of delivery, only women who ultimately delivered when the laboratory and research assistants were available could participate. The first 100 women to consent to participation and to meet eligibility requirements were enrolled. This study was approved by the Western Institutional Review Board.

Before hospital discharge, participants were asked to complete a questionnaire that recorded demographic information and a dietary survey of seafood consumption during the last month of pregnancy. Participants reported their own race, and the race or races of their parents and grandparents. For analysis, participants were categorized into a race if they identified themselves as at least part of that race. Women were asked to quantify the amount of seafood they had eaten by recording how many times they had eaten seafood in the last month and how many ounces they typically eat in a setting (they were told that 3 oz is approximately the size of a deck of cards). Total fish consumption in the last month was calculated by multiplying the number of times the women reported eating seafood by the number of ounces of seafood they typically eat at each meal. Women also indicated the types of seafood they consumed in the last month, and the number of times they ate each fish.

After delivery a single tube of approximately 14 mL of whole blood from the umbilical cord was collected into a standard laboratory issued EDTA tube. All samples were processed within two hours of delivery and stored at -25o C. Samples were sent to the Energy and Environmental Research Center (Grand Forks, North Dakota), and were analyzed for mercury content by cold-vapor atomic absorption spectrophotometry using a CETAC M-6000A (CETAC Technologies, Omaha, NE).

Descriptive statistics were performed to assess differences in sociodemographic factors among women at different levels of seafood consumption. Continuous values (e.g. mercury concentrations) were transformed to log values prior to analysis to account for non-normal distribution. The magnitude and significance of association between fish consumption, umbilical cord blood mercury concentrations and sociodemographic factors was calculated using chi square tests for categorical variables and analysis of variance for continuous variables. Multiple logistic regression was utilized to confirm the association and significance of relationships using odds ratios for marginally-associated variables with fish consumption and mercury concentrations (p < .10) as potential confounding variables for inclusion in the model. All analyses were performed using SPSS 20.0 (IBM SPSS Statistics for Mac, Version 20.0. Armonk, NY: IBM Corp). Results are not presented for population subgroups of less than ten women.

## Results

A total of 107 women consented to participate and initially met eligibility requirements. Two women withdrew consent prior to being discharged from the hospital, and two women were withdrawn because they could not read the English-language consent form. As part of the primary study, all neonates underwent a specialized neonatal developmental exam prior to their discharge from the hospital. Three women were withdrawn because they were discharged prior to their infants’ neonatal exam. Once 100 women met all eligibility requirements, enrollment for this pilot study stopped. The primary ethnicities of the birthing population of Hawaii are represented in the sample, though comparing precise representation is difficult since this study allowed multiple ethnicity reporting and statewide databases typically report race and ethnicity using other algorithms [22]. The most commonly reported ethnicities in our study (Hawaiian and Pacific Islander, Caucasian, Filipino, and Japanese) reflect the same ethnicities and order found in the birthing population of Hawaii [23].

The mean age of the women was 28.3 years (range 18–44 years), with a mean gravidity of 2.93 (range 1–9) and parity 1.38 (range 0–6). Participants came from a variety of racial backgrounds, with 54% of participants identifying themselves as two or more races, and 36% identifying with three or more racial groups.

Most women (86%) reported eating seafood during the previous month, with 9% eating more than the recommended limit during pregnancy. Women who self- identified as at least part Filipino reported consuming less seafood than their non-Filipina counterparts (p < .05). Women of most ethnicities consumed similar quantities of fish, with the exception of Japanese women, who consumed significantly less fish than non-Japanese women, and Pacific Islander women (includes Native Hawaiian, Samoan, Tongan, and Micronesian), who consumed significantly more (Table 1).

**Table 1:** Demographic characteristics, fish consumption and MeHg concentrations in umbilical cord blood

	**Total n**	**Ate fish (%)**	**Mean ounces of fish consumed in last month of pregnancy (+/- SD)**	**MeHg concentration in cord blood ≥5 μg/L**
Total	100	86.0 (86)	19.8 (3.7)	44.0 (44)
Race**				
Hispanic	18	88.9 (16)	18.8 (15.0)	33.3 (6)
Caucasian	42	88.1 (37)	18.3 (18.8)	42.9 (18)
Japanese	23	91.3 (21)	9.6 (7.4)	56.5 (13)
Chinese	23	82.6 (19)	16.2 (20.0)	34.8 (8)
Filipino	32	75.0 (24)*	13.7 (15.0)	28.1 (9)*
Pacific Islander¶	48	87.5 (42)	25.7 (34.8)*	43.8 (21)
Birthplace				
United States	65	81.5 (53)	17.4 (20.7)	38.5 (25)
Outside of United States	34	94.1 (32)	23.5 (37.9)	52.9 (18)
Income				
Less than $22,000	20	95.0 (19)*	38.0 (47.7)*	50.0 (10)
$22,000–$47,600	21	71.4 (15)	12.2 (18.5)	38.1 (8)
$47,600–$80,100	22	86.4 (19)	22.5 (23.0)	36.4 (8)
Greater than $80,100	19	89.5 (17)	13.3 (11.1)	47.4 (9)
Missing	18	88.9 (16)	11.9 (13.3)	50.0 (9)
Highest education level achieved				
Less than high school	9	88.9 (8)	32.8 (35.8)	55.6 (5)
High school diploma	39	84.6 (33)	22.8 (36.2)	46.2 (18)
Any college	52	86.5 (45)	15.2 (16.1)	40.4 (21)
Mother’s age				
Less than 23 years	27	77.8 (21)	18.3 (21.9)	33.3 (9)
23 – 27.5 years	23	87.0 (20)	24.7 (31.7)	47.8 (11)
27.5 – 33 years	26	92.3 (24)	19.0 (35.6)	38.5 (10)
Over 33 years	24	87.5 (21)	17.6 (20.2)	58.3 (14)

*p < .05.

**Numbers add up to more than 100 because women could identify with more than one racial group (n = 54).

¶Pacific Islander includes Native Hawaiian, Samoan, Tongan, and Micronesian.

The median household income reported by participants was $47,500. Women in the lowest income category, under $22,000 per year, ate significantly more fish in the last month of pregnancy than women in the other income categories, and 30% of them reported consumption of more than the recommended 12 ounces per week. Fish consumption did not vary by women’s age, education, or whether or not they were born in the United States (Table 1).

The mean mercury concentration in umbilical cord blood was 5.2 μg Hg/L. Almost half (44%) of all women had mercury concentrations of 5 μg Hg/L or more. Filipino women were significantly less likely to have elevated Hg levels when compared with non-Filipino women (p < .05). Mercury levels did not vary by other demographic characteristics (Table 1).

**Figure 1:**
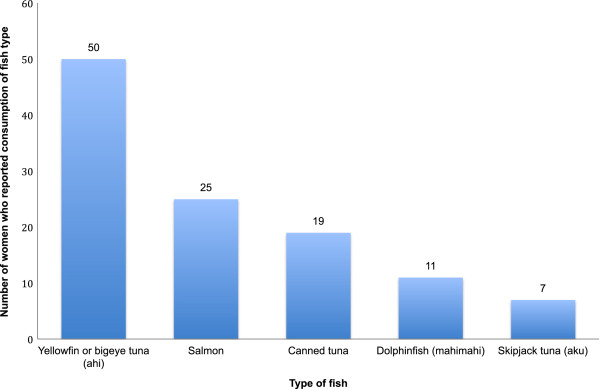
Top five types of fish consumed in pregnancy, Hawaii cohort.

Women who reported eating more than the recommended 12 ounces of fish per week were significantly more likely to have umbilical cord blood concentrations of 5 μg Hg/L or more, but mean mercury concentrations were not significantly higher (6.1 ± 3.3 v 5.0 ± 3.7). Among women who reported eating any fish as compared with women who reported no fish consumption, however, the odds ratio for having a cord blood mercury concentration of 5 μg Hg/L or more was significantly higher (5.7; 95% confidence interval: 1.2, 27.1) (Table 2). The most commonly consumed fish (Figure 1) was yellowfin or bigeye tuna (ahi), nearly twice as common as the next most commonly consumed fish (salmon).

**Table 2:** Fish consumption and MeHg concentrations in cord blood

	**Percent of women with mean Hg ≥ 5 μg/L (#)**	**Mean Hg concentration in μg/L (+/- SD)**
Total	44.0 (44)	5.2 (3.7)
Fish consumption		
More than 12 oz/week	77.8 (7)*	6.1 (3.3)
0–12 oz/week	40.0 (36)	5.0 (3.7)
Any fish consumed	48.8 (42)*	5.6 (3.8)*
No fish consumed	14.3 (2)	2.3 (1.8)

*p < .05.

## Discussion

The majority of women in this study consumed seafood during the last month of pregnancy, and 9% averaged more than the recommended limit of 12 ounces per week. This finding was consistent with a previous study conducted in Hawaii that found that 94% of the participants had consumed fish at some point during their pregnancy and 19% reported eating seafood more than two times a week, although researchers did not quantify how much seafood was consumed nor whether ethnicity or income were associated with either fish consumption or serum mercury concentrations [21]. In our study, fish consumption varied by ethnicity and income, which is an important contribution to our understanding of patterns. This study’s findings are consistent with statewide surveillance of fish consumption among adults reported by the Hawaii State Health Department [24].

In the 1999–2000 National Health and Nutrition Examination Survey (NHANES), 8% of reproductive-aged women nationwide reported consuming more than the recommended amount of seafood [25]. Based on this estimate, one analysis calculated that approximately 300,000 infants born every year are at risk for learning disabilities and other neurodevelopmental effects due to in utero exposure to mercury [26]. A subsequent sub-analysis of the 1999–2000 NHANES reported that 16.6% of women who identified as Asian, Pacific Islander, Native American, or multiracial had blood mercury levels of 5.8 μg/L or greater, as compared to 5.1% of the remaining participants [27]. In this sample of 140 women, the average number of seafood meals was also higher than in the other groups, although this was not a statistically significant difference. A state like Hawaii with a large multiracial, and particularly inclusive of Asian and Pacific Islander, population would expect similarly higher mercury concentrations.

Our study findings are limited by the small number of participants. While, overall, a study sample of 100 with a moderate effect size is sufficient for a pilot study [28], subgroup analyses by ethnicity and other demographic characteristics could be inaccurate. Moreover, the sample was not stratified in recruitment by ethnicity, thus subpopulations are not necessarily representative. Additionally, race and ethnicity reporting in a multiethnic state challenges traditional reporting mechanisms, though in this study that we allowed for women to self-identify multiple ethnicities should decrease misclassification bias [22]. Further, though food frequency methods are often used in assessing fish consumption in similar studies [17], fish consumption could be misclassified if recall of typical consumption is biased. Finally, these results are from a secondary analysis of a primary pilot study that aimed to investigate the interactions of methylmercury, selenium and omega-three fatty acids in fetal neurodevelopment; participants are self-selected and not randomly sampled, and therefore may not represent the general population of pregnant women in Hawaii.

## Conclusion

In the 2003–2008 NHANES, mean mercury blood concentration among pregnant women was 0.69 μg/L [29]. In our study, the mean umbilical cord blood mercury concentration was over seven times higher at 5.2 μg Hg/L, with values ranging from a low of 0.4 μg/L to a high of 22.5 μg/L. Taking into account estimates that the mercury concentration in cord blood is 1.7 times higher than in maternal blood [30], mercury concentrations in our study were still over four times higher than those in the most recent NHANES analysis. Despite EPA guidelines, a significant portion of pregnant women consumed more than the recommended amount of seafood, which was associated with race and income. Further, almost half of study participants had cord blood Hg concentrations at or exceeding 5 μg/L.

In 2000, the NRC issued a public health directive to investigate fish consumption in various subpopulations to identify those at higher risk for adverse effects [11]. Clearly, subpopulations of pregnant women consume more seafood than others and, therefore, have higher concentrations of mercury in their umbilical cord blood. What remains unanswered is whether the methylmercury exposure typically experienced by most people, including the subpopulations of women identified in this study and others, results in any adverse fetal effects. This study suggests that better understanding of fish consumption patterns in pregnancy, ethnic variation, and public health impact is required, particularly in multiethnic states like Hawaii, to optimize health benefits of fish consumption while reducing risk.

## Abbreviations

EPA: Environmental Protection Agency; NRC: National Research Council; BMDL: Benchmark Dose Lower Limit; FDA: Food and Drug Administration; NHANES: National Health and Nutrition Examination Survey.

## Competing interests

The authors disclose no competing interests regarding this study.

## Authors’ contributions

RS designed the study, collected specimens, and drafted the manuscript. TD performed the statistical analyses and helped draft the manuscript. NR carried out the assays. MB and LS participated in the design of the study. All authors read and approved the final manuscript.

## Pre-publication history

The pre-publication history for this paper can be accessed here:

http://www.biomedcentral.com/1471-2393/14/209/prepub
